# Can Scoliosis Help the Early Diagnosis of Congenital Myasthenic Syndrome?

**DOI:** 10.7759/cureus.45875

**Published:** 2023-09-24

**Authors:** Oğuz Kaya, Serkan Kirik

**Affiliations:** 1 Orthopaedics and Traumatology, Elazığ Fethi Sekin City Hospital, Elazığ, TUR; 2 Pediatric Neurology, Fırat University School of Medicine, Elazığ, TUR

**Keywords:** ptosis, colq, pediatrics, congenital myasthenic syndromes, scoliosis

## Abstract

Background

Congenital myasthenic syndromes (CMS) are a group of hereditary diseases of the neuromuscular junction. CMS are extremely rare diseases that cause hypotonia; however, scoliosis may theoretically be helpful in early diagnosis of CMS. The objective of this study was to emphasize the clinical features of the patients we followed up with the diagnosis of CMS and demonstrate that scoliosis is an important finding in the diagnosis of CMS in the presence of hypotonia/weakness.

Materials and methods

In this retrospective study, data were retrieved by examining the digital files of the patients who presented to Aydın Maternity and Children’s Hospital and Elazığ Fethi Sekin City Hospital Pediatric Neurology Clinics between 2018 and 2023. The diagnosis of CMS was strongly supported by a combination of clinical characteristics, neurophysiological studies, genetic tests, AChR antibodies, and serum creatine kinase measurement. The presence of scoliosis was evaluated by an orthopedics and traumatology specialist.

Results

Eleven CMS patients with accompanying scoliosis were included in the study. The mean age of the patients was 69.4±39.28 months. The age of the patients at the time of diagnosis was 42.7±35.19 months. Among the patients, eight were males (72.7%), and three were females (27.2%). Seven patients (63.6%) had COLQ mutations. Electromyography was conducted on eight patients, with one of them showing no pathological findings, while seven exhibited decremental responses. All patients had ptosis, while six (54.5%) had bulbar signs. Ten patients (90.9%) had weakness. Nine patients (81.8%) experienced frequent recurrent lower respiratory tract infections. Both the patient with CHAT mutation and RAPSN mutation had arthrogryposis.

Conclusion

In this study, CMS stands out as an essential consideration in the differential diagnosis, particularly when scoliosis accompanies early-onset muscle weakness.

## Introduction

Congenital myasthenic syndromes (CMS) are a heterogeneous group of diseases within the category of childhood neuromuscular disorders. Unlike other myasthenic diseases, CMS do not have an immune origin. The condition leads to a wide range of clinical manifestations, spanning from mild muscle weakness and scoliosis to severe respiratory failure, which complicates the diagnostic process [1]. CMS is caused by structural defects in various synaptic proteins involved in neuromuscular transmission, resulting from different mutations. The annual incidence of CMS in Western countries has been reported to be 30/1000000. Specifically, among individuals under the age of 20, the incidence is estimated to be between 1 and 5 cases per million, with symptoms starting at the age of 14 or earlier in 44.8% of cases. In recent years, whole exome sequencing (WES) has helped in diagnosing new CMS cases, leading to an increase in the number of diagnosed cases [2-4]. Furthermore, the expanded availability of CMS panels has helped identify numerous CMS-related genes. Depending on the location of the mutant protein, CMS is divided into three subtypes: presynaptic, synaptic basal lamina-associated, and postsynaptic [3,5].

In this study, we aimed to emphasize the importance of scoliosis as an important early indicator of congenital myasthenic syndrome in aiding diagnosis and to increase awareness by presenting 11 cases with scoliosis among 17 patients followed up with the diagnosis of CMS.

## Materials and methods

In this retrospective study, the data were retrieved by examining the digital files of the patients who presented to Aydın Maternity and Children’s Hospital and Elazığ Fethi Sekin City Hospital Pediatric Neurology Clinics between 2018 and 2023. Seventeen pediatric patients who were genetically diagnosed with CMS and had scoliosis were included in this study. However, six patients who did not have a genetic diagnosis, whose digital data could not be accessed, and who did not have any signs of scoliosis were excluded from the study.

The diagnosis of CMS was strongly supported by a combination of clinical characteristics, neurophysiological studies, and laboratory investigations, including AChR antibodies and serum creatine kinase measurement. After evaluating the clinical diagnosis in all our cases, the genetic diagnosis was made utilizing WES or the CMS panel. We examined the presence of scoliosis and recorded the age of onset based on patient files. Venous blood samples were obtained from patients and their unaffected relatives for DNA extraction. Genomic DNA isolation was conducted according to the manufacturer's recommendations using a blood DNA extraction kit from Promega, Germany.

Written informed consent was obtained from all patients before data collection. Approval from the local ethics committee was also obtained (Date: 24.05.2022, Decision No: 2022/5-27).

Statistical analysis

Data obtained were evaluated using the Statistical Product and Service Solutions (SPSS) (version 22.0; IBM SPSS Statistics for Windows, Armonk, NY) program. Descriptive statistics included categorical variables presented as numbers and percentages, while numerical variables were described using mean, standard deviation, minimum, maximum, and median values.

## Results

**Figure 1:**
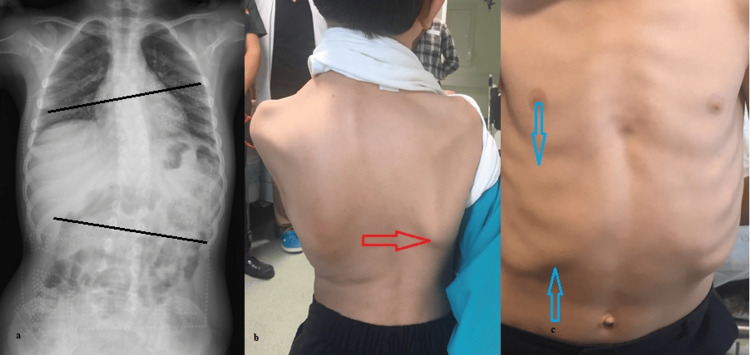
a. Cobb angle measurement 20.3 degrees. b. Curve of spinous processes due to scoliosis is observed and elevation of the right scapula due to scoliosis (red arrow). c. Inequality of chest and rib deformity observed (blue arrows)

Eleven CMS patients with accompanying scoliosis were included in the study. The mean age of the patients was 69.4±39.28 months. The age of the patients at the time of diagnosis was 42.7±35.19 months. Age, gender, gene, and clinical findings are detailed in Table 1. Among the patients, eight were males (72.7%), and three were females (27.2%). Seven patients (63.6%) had COLQ mutations, two (18.1%) had CHAT mutations, one (0.09%) had RAPSN mutation, and one (0.09%) had DOK7 mutation. Electromyography was conducted on eight patients, with one of them showing no pathological findings, while seven exhibited decremental responses. Anti-MuSK and acetylcholinesterase tests were performed on all patients, all yielding negative results. All patients had ptosis, while six (54.5%) had bulbar signs. Ten patients (90.9%) had weakness, and one (0.09%) was on continuous mechanical ventilation with a tracheostomy. Except for the patient on mechanical ventilation, respiratory functions were normal between attacks in other patients. Nine patients (81.8%) experienced frequent recurrent lower respiratory tract infections. Both the patient with CHAT mutation and RAPSN mutation had arthrogryposis. The radiological and clinical findings of the patients are shown in Figure 1.

**Table 1:** Gender, age, genetic, and additional clinical findings of patients

	Gender	Age at diagnosis (Months)	Current age (Months)	Gene	Additional findings
1	Male	13	39	COLQ	
2	Male	32	43	COLQ	Mechanical ventilation
3	Female	23	37	COLQ	
4	Male	34	65	COLQ	
5	Male	21	52	COLQ	
6	Male	147	207	COLQ	
7	Male	6	76	COLQ	
8	Female	118	155	CHAT	Arthrogryposis
9	Male	32	46	CHAT	
10	Female	21	40	RAPSN	Arthrogryposis
11	Male	14	36	DOK7	

All patients carrying DOK7 and COLQ mutations were started on ephedrine, resulting in favorable responses. Significant regression in muscle weakness was observed in patients receiving ephedrine treatment (Figure 1).

## Discussion

CMS is a genetic, heterogeneous neuromuscular disorder characterized by abnormalities in the synaptic transmission system. Congenital myasthenic syndromes are rare and challenging to diagnose, with varying clinical findings [1,6,7]. Unlike myasthenia gravis, the syndromes are not autoimmune, do not respond to immunosuppressive therapy, and cannot be diagnosed by antibodies. Several different gene mutations have been identified in CMS, including CHAT, COLQ, CHRNA1, CHRNB1, CHRND, CHRNE, RAPSN, MUSK, DOK7, and SCN4A. CMS is classified as presynaptic, synaptic, or postsynaptic, depending on the location of the defect, resulting in differing clinical presentations. With the increasing accessibility of WES and targeted gene panels, these methods have become more prevalent in CMS diagnosis in recent years [6-8]. In our study, the diagnosis of three patients was achieved through WES analysis, while the diagnosis of three others was made using a gene panel.

Skeletal deformities, such as scoliosis, are more commonly encountered in syndromes with DOK7 mutations, typically manifesting as normal early motor development and eye movement after the age of two. The deformities are less common in cases with COLQ mutations. Over time, fluctuating scoliosis due to truncal muscle weakness progresses into severe scoliosis. This is particularly evident in patients with DOK7 and COLQ mutations [5,7-9]. Della Marina et al. reported that two of three patients with COLQ mutations developed scoliosis, with one of them requiring surgical intervention [8]. Similar to the adult population, scoliosis may be one of the clinical symptoms specific to AChE deficiency, as previously suggested [10]. A very specific indicator of synaptic mutation is the worsening of symptoms during treatment with AChE inhibitors [11,12]. Most CMS patients respond well to treatment with acetylcholinesterase inhibitors. However, in cases with DOK7 and COLQ mutations and slow-channel congenital myasthenic syndrome, symptoms worsen due to pyridostigmine treatment. Ephedrine and salbutamol have been used successfully in patients with COLQ and DOK7 mutations [12,13]. In our study, patients with COLQ mutation were in the majority, and these patients were receiving ephedrine treatment.

The most important limitation of our study was the limited number of cases from a small number of centers. Future research will benefit from multicenter studies with a larger patient population.

## Conclusions

In conclusion, CMS can lead to early-onset and severe muscle weakness. It is known that fatal complications and hospitalizations are reduced in cases diagnosed in the early period. In this study, CMS stands out as an essential consideration in the differential diagnosis, particularly when scoliosis accompanies early-onset muscle weakness.
